# Neutrophilia and post-radiation thrombocytopenia predict for poor prognosis in radiation-treated glioma patients

**DOI:** 10.3389/fonc.2022.1000280

**Published:** 2022-09-09

**Authors:** Eric J. Hsu, Jamie Thomas, Elizabeth A. Maher, Michael Youssef, Robert D. Timmerman, Zabi Wardak, Minjae Lee, Tu D. Dan, Toral R. Patel, Dat T. Vo

**Affiliations:** ^1^ Department of Radiation Oncology, UT Southwestern Medical Center, Dallas, TX, United States; ^2^ Department of Neurological Surgery, UT Southwestern Medical Center, Dallas, TX, United States; ^3^ Department of Internal Medicine, Division of Hematology and Oncology, UT Southwestern Medical Center, Dallas, TX, United States; ^4^ Department of Neurology, UT Southwestern Medical Center, Dallas, TX, United States; ^5^ Department of Population and Data Sciences, UT Southwestern Medical Center, Dallas, TX, United States

**Keywords:** radiation therapy, glioma, lymphopenia, neutrophilia, thrombocytopenia, neutrophil-to-lymphocyte ratio, platelet-to-lymphocyte ratio, OS/PFS

## Abstract

**Introduction:**

Poor outcomes in glioma patients indicate a need to determine prognostic indicators of survival to better guide patient specific treatment options. While preoperative neutrophil-to-lymphocyte ratio (NLR), platelet-to-lymphocyte ratio (PLR), and monocyte-to-lymphocyte ratio (MLR) have been suggested as prognostic systemic inflammation markers, the impact of post-radiation changes in these cell types is unclear. We sought to identify which hematologic cell measurements before, during, or after radiation predicted for patient survival.

**Methods:**

A cohort of 182 patients with pathologically confirmed gliomas treated at our institution was retrospectively reviewed. Patient blood samples were collected within one month before, during, or within 3 months after radiation for quantification of hematologic cell counts, for which failure patterns were evaluated. Multivariable cox proportional hazards analysis for overall survival (OS) and progression-free survival (PFS) was performed to control for patient variables.

**Results:**

Multivariable analysis identified pre-radiation NLR > 4.0 (Hazard ratio = 1.847, p = 0.0039) and neutrophilia prior to (Hazard ratio = 1.706, p = 0.0185), during (Hazard ratio = 1.641, p = 0.0277), or after (Hazard ratio = 1.517, p = 0.0879) radiation as significant predictors of worse OS, with similar results for PFS. Post-radiation PLR > 200 (Hazard ratio = 0.587, p = 0.0062) and a percent increase in platelets after radiation (Hazard ratio = 0.387, p = 0.0077) were also associated with improved OS. Patients receiving more than 15 fractions of radiation exhibited greater post-radiation decreases in neutrophil and platelet counts than those receiving fewer. Patients receiving dexamethasone during radiation exhibited greater increases in neutrophil counts than those not receiving steroids. Lymphopenia, changes in lymphocyte counts, monocytosis, MLR, and changes in monocyte counts did not impact patient survival.

**Conclusion:**

Neutrophilia at any time interval surrounding radiotherapy, pre-radiation NLR, and post-radiation thrombocytopenia, but not lymphocytes or monocytes, are predictors of poor patient survival in glioma patients.

## Introduction

Gliomas are the most common primary intracranial tumor, accounting for more than 80% of all malignant brain tumors (1). Glioblastoma, the most common type of glioma, affects 3.23 persons per 100,000 in the United States every year (2). Management of glioblastoma entails maximally safe surgical resection followed by adjuvant radiation therapy with concomitant and adjuvant temozolomide, whereas management of lower grade gliomas includes surgical resection followed by risk-adjusted adjuvant radiation therapy and adjuvant chemotherapy, with the treatment paradigm constantly evolving (3). In recent years, the addition of tumor-treating fields has improved glioblastoma survival to up to a median time of 20.9 months (4, 5). For patients with gliomas, there are varying outcomes based on histology and molecular status with patients with glioblastoma still exhibit a 5 year survival of ~5% after initial diagnosis, whereas patients with grade 2 oligodendroglioma have a median survival of 13 years (6, 7). These outcomes in glioma patients indicate a need to determine prognostic indicators of survival to better guide patient specific treatment options.

Numerous studies have evaluated the prognostic significance of hematologic cell counts in patients with gliomas, often in the form of preoperative systemic cellular inflammatory markers. Observed markers include neutrophil-to-lymphocyte ratio (NLR), platelet-to-lymphocyte ratio (PLR), and monocyte-to-lymphocyte ratio (MLR) or lymphocyte-to-monocyte ratio (LMR) (8). However, despite multiple retrospective studies and systematic reviews, no consensus has been reached for which markers, especially those related to platelets and monocytes, consistently predict for poor prognosis (8–11). Furthermore, the prognostic role of absolute hematologic cell counts, especially those in the intra- or post-treatment time intervals, is not well understood in glioma patients. Treatments and medications involved in the care of patients with gliomas can significantly alter hematologic cell counts. Patients who have received radiotherapy may exhibit significant radiation induced decreases in systemic immune cell profiles after treatment (12). In particular, radiation induces a decrease in lymphocytes, neutrophils and platelets but has not been observed to affect monocyte counts (13, 14). Similarly, cytotoxic chemotherapy on the bone marrow has also been observed to reduce lymphocyte, neutrophil, and platelet but not monocyte counts (15–17). Lastly, steroid induced immunosuppression can cause a decrease in lymphocytes and increase in neutrophils (18, 19). Therefore, this study investigates which systemic inflammation marker ratios, absolute lymphocyte, neutrophil, platelet, and monocyte counts, and percent changes in cell counts at pre-, intra-, and post-radiation time intervals can predict for survival in patients with gliomas.

## Materials and methods

A database of 182 patients with primary brain gliomas treated with radiation therapy at our institution between October 2010 and December 2021 was retrospectively reviewed. Patients underwent pathological typing according to the 2021 WHO Classification of Tumors of the Central Nervous System (20). The study was approved by the UT Southwestern institutional review board (IRB number STU 062014-027).

All patients received radiation therapy targeted to either the primary tumor or the post-tumor resection cavity, with patients receiving doses ranging from 20 to 75 Gy in 5 to 33 fractions. Patients underwent CT simulation with a tailored head-thermoplastic mask in the supine position. A gross tumor volume (GTV) is delineated using a fused postoperative MRI on the T1 and T2 FLAIR sequences, followed by a creation of a clinical target volume (CTV) to cover the potential areas of microscopic disease. Then, a planning target volume (PTV) expansion was created to account for daily uncertainty in daily set-up and treatment delivery, per our institutional protocol and standards.

We then assessed the patterns of failure, including in-field failures (within the 95% isodose volume), out-of-field failures, or marginal failures (within the 50-95% isodose volume) as observed radiographically on MRI. Time to recurrence was defined as the time from the end of the radiation treatment period to the first radiographic evidence of recurrence.

### Cell quantification and measurement

The pre-radiation time interval was defined as 1 month prior to the start of radiation therapy. The intra-radiation time interval was defined as between the first and final sessions of radiation therapy. The post-radiation time interval was defined as 3 months after the end of radiation therapy. Lymphopenia was defined as any recorded lymphocyte count less than 1,060 lymphocytes per μL in a specific time interval. Neutrophilia was defined as any recorded neutrophil count greater than 8,500 neutrophils per μL in a specific time interval. Thrombocytopenia was defined as any recorded platelet count less than 150,000 platelets per μL in a specific time interval. Monocytosis was defined as any recorded monocyte count greater than 900 monocytes per μL in a specific time interval. Absolute cell counts during each time interval were calculated as the average of all recorded cell counts during that time interval. Intra-radiation percent changes in cell counts were calculated by taking the difference between absolute cell counts during the radiation time interval and pre-radiation time interval and then dividing by the absolute cell counts during the pre-radiation time interval. Post-radiation percent changes were calculated similarly but during the post-radiation time interval instead.

### Statistics

Overall survival (OS) and progression-free survival (PFS) were estimated using Kaplan-Meier method. Patients who were alive without evidence of recurrence were censored at the date of last follow up. P values were calculated from incidence of recurrence or death and survival curves were created with Cox proportional hazards tests. P values were considered significant at < 0.05.

Cox proportional hazards regression was used to determine the impact of patient covariates on PFS and OS. Hazard ratios and confidence intervals were calculated for each variable. Multivariable Cox proportional hazards regression models were used to adjust for patient characteristics (number of radiation fractions, age, body mass index (BMI), gender, p53 mutation status, MGMT methylation status, presence of edema, and presence of seizures) for each multivariable analysis.

## Results

Of the 182 patients included in the analysis, the median age of all patients at the time of initiation of radiation therapy was 57.0 years. Gender, histology, mutation status, presence of edema at initiation of radiation, and presence of seizures are detailed in **Table 1**. Patients were treated with radiation therapy in 5 to 33 fractions, with increasing fractions corresponding to increased total radiation dose prescribed. Patients who received temozolomide or dexamethasone during radiation therapy were numbered at 46.2% and 27.5%, respectively. Median follow-up for all patients was 16.8 months (range = 0.3 to 109.9 months).

**Table 1:** Patient characteristics.

Characteristics	Number of patients (%)
Total Patients	182
Age (years)	
Median	57.0
Range	18.8 – 79.5
BMI	
Median	27.8
Range	14.9 – 48.8
Gender	
Male	112 (61.5%)
Female	70 (38.5%)
Histology	
Glioblastoma Multiforme	126 (69.2%)
Other Astrocytoma	33 (18.1%)
Oligodendroglioma	23 (12.6%)
IDH Status	
Wild Type	139 (76.4%)
Mutant	43 (23.6%)
P53 Status	
Wild Type	163 (89.6%)
Mutant	19 (10.4%)
MGMT Status	
Wild Type	162 (89.0%)
Methylated	20 (11.0%)
Edema	
Present	101 (55.5%)
Absent	81 (44.5%)
Seizures	
Present	115 (63.2%)
Absent	67 (36.8%)
Concurrent Temozolomide	
Present	84 (46.2%)
Absent	98 (53.8%)
Concurrent Dexamethasone	
Present	50 (27.5%)
Absent	132 (72.5%)
Treatment Duration (fractions)	
Median	25
Range	5 – 33
Follow Up Duration (months)	
Median	16.8
Range	0.3 – 109.9

BMI, Body Mass Index; IDH, Isocitrate Dehydrogenase; MGMT, 06-Methylguanine-DNA Methyltransferase; CI, Confidence Interval.

**Figure 1:**
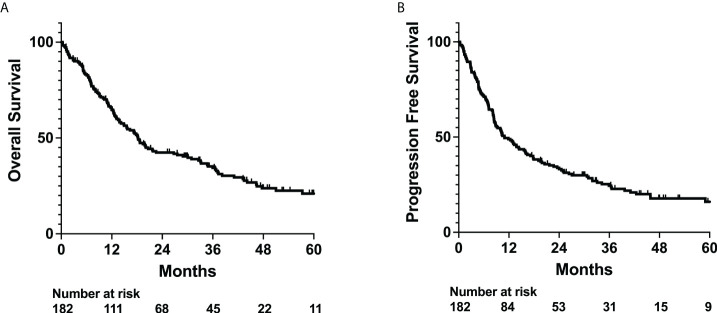
OS and PFS outcomes for whole patient cohort. Kaplan-Meyer plot of **(A)** overall survival (OS) and **(B)** progression free survival (PFS).

For the entire cohort, median OS and PFS were 18.2 and 11.0 months (**Figure 1**), respectively. Univariate analysis of patient characteristics was performed to determine predictors of OS and PFS (**Supplementary Table 1**). Number of radiation fractions, age, and BMI were analyzed as continuous variables. Increased number of fractions was associated with improved OS (Hazard Ratio 0.924, 95% CI 0.902 to 0.947) and PFS (Hazard Ratio 0.931, 95% CI 0.909 to 0.953). Increased age was associated with worse OS (Hazard Ratio 1.038, 95% CI 1.024 to 1.052) and PFS (Hazard Ratio 1.030, 95% CI 1.017 to 1.043). Gender, IDH mutation status, p53 mutation status, MGMT methylation status, presence of edema, presence of seizures, concurrent temozolomide treatment, and concurrent dexamethasone use were analyzed as categorical variables. Mutant IDH status was strongly associated with improved OS (Hazard Ratio 0.166, 95% CI 0.089 to 0.310) and PFS (Hazard Ratio 0.166, 95% CI 0.093 to 0.296). Dexamethasone use during radiation was associated with worse OS (Hazard Ratio 1.669, 95% CI 1.148 to 2.484) and PFS (Hazard Ratio 1.652, 95% CI 1.147 to 2.379). Wild type p53 status and presence of edema were either statistically significantly associated or trended towards worse OS and PFS.

Lymphopenia has been correlated with recurrence and poor outcomes in multiple types of cancer. We performed univariate and multivariable analysis to determine how lymphopenia affects patient outcomes. While lymphopenia before and after radiation appeared to have statistically significant effects on OS as a standalone variable, multivariable analysis suggests that lymphopenia does not statistically significantly affect OS or PFS (**Supplementary Figure 1**; **Supplementary Tables 2, 3**).

While the hematologic states of patients during specific time points may be predictive prognostic factors, they do not account for potential changes in cell counts from before treatment to during or after treatment. In this context, we evaluated how changes in lymphocyte counts could potentially impact survival. A percent increase in lymphocyte count from before to after radiation treatment was associated with improved OS on univariate analysis but not multivariable analysis (**Supplementary Tables 2, 3**). As a higher number of fractions of radiation are associated with improved outcomes (**Supplementary Table 1**) and radiotherapy has cytotoxic effects on immune cells, we also assessed whether number of fractions impacted lymphocyte counts in our patient population (12). We observe that patients who received more than 15 fractions of radiation exhibited a larger percent decrease and lower absolute count of lymphocytes after radiation treatment (**Supplementary Figure 2**). Similarly, chemotherapy or steroid use can also affect lymphocyte counts. We observed temozolomide use during radiation treatment did not affect lymphocyte counts, but dexamethasone use was significantly associated with a greater percent decrease in lymphocytes (**Supplementary Figures 3A, B, 4A, B**).

**Figure 2:**
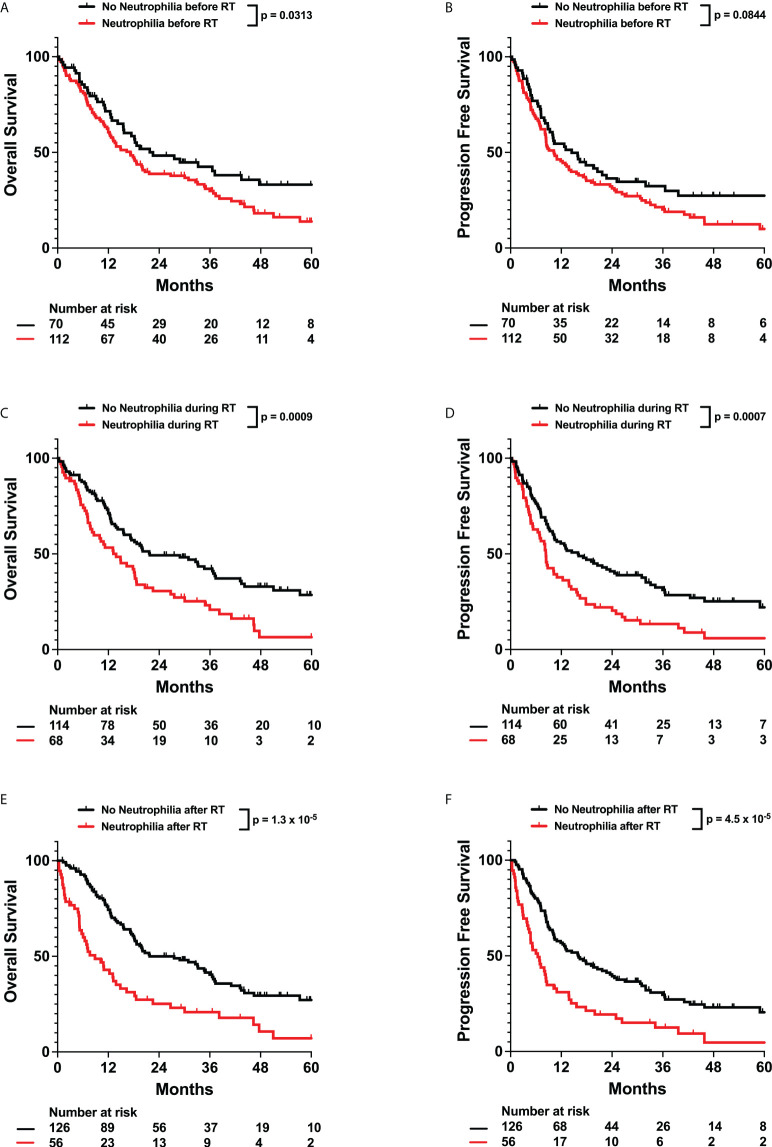
Effects of Neutrophilia on OS and PFS. Kaplan-Meyer plots of OS and PFS comparing patients with neutrophilia **(A, B)** before, **(C, D)** during, **(E, F)** or after radiation treatment. Statistical analysis was performed using Cox Proportional Hazards tests. OS, Overall Survival; PFS, Progression Free Survival; RT, Radiation therapy.

**Figure 3:**
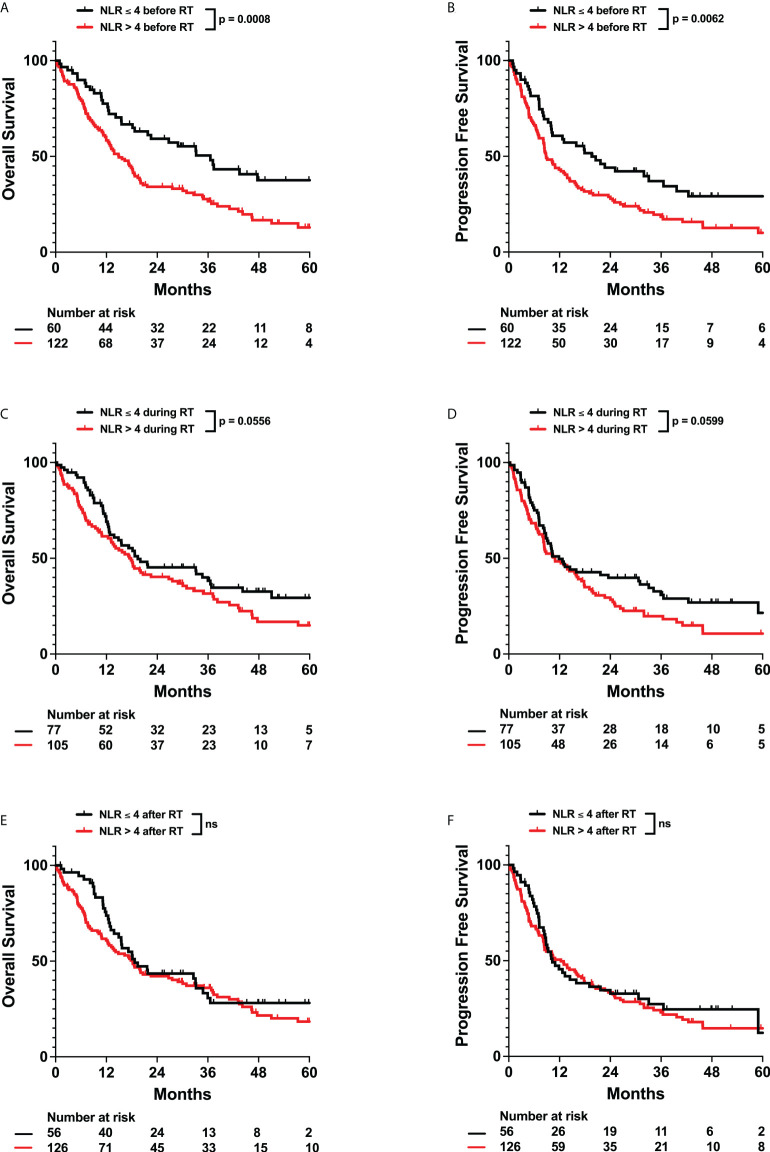
Effects of Neutrophil to Lymphocyte Ratio (NLR) on OS and PFS. Kaplan-Meyer plots of OS and PFS comparing patients with NLR > 4 **(A, B)** before, **(C, D)** during, **(E, F)** or after radiation treatment. Statistical analysis was performed using Cox Proportional Hazards tests. OS, Overall Survival; PFS, Progression Free Survival; NLR, Neutrophil to Lymphocyte Ratio; RT, Radiation therapy.

**Figure 4:**
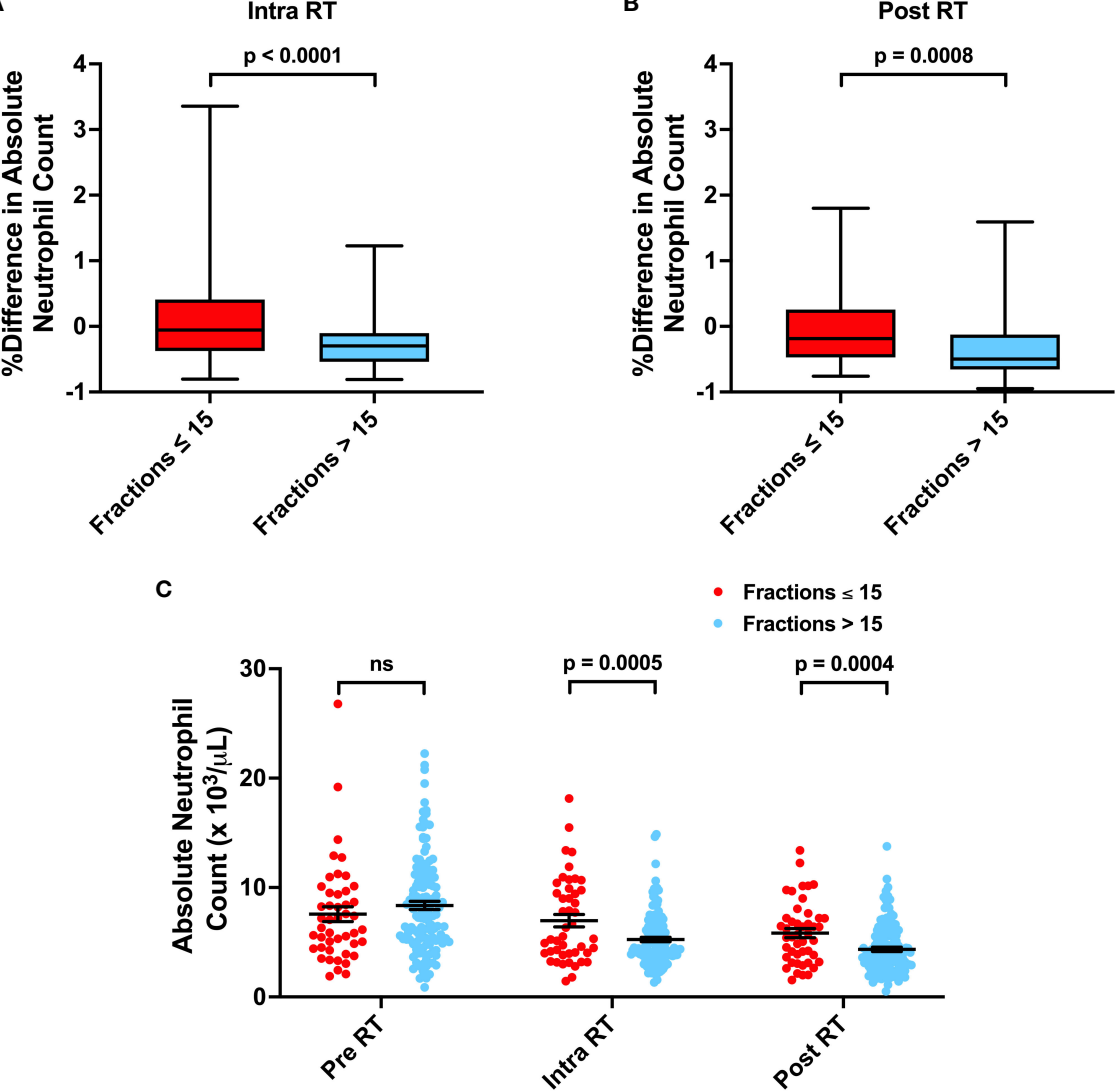
Associations between Number of Radiation Fractions and Neutrophil Counts. **(A, B)** Percent difference in neutrophil counts for patients receiving ≤ 15 or > 15 fractions from before radiation treatment (RT) to during RT or after RT. **(C)** Absolute neutrophil counts before, during, and after RT for patients receiving ≤ 15 or > 15 fractions. Statistical analysis was performed using two sample t-tests. ns, not significant.

**Figure 5:**
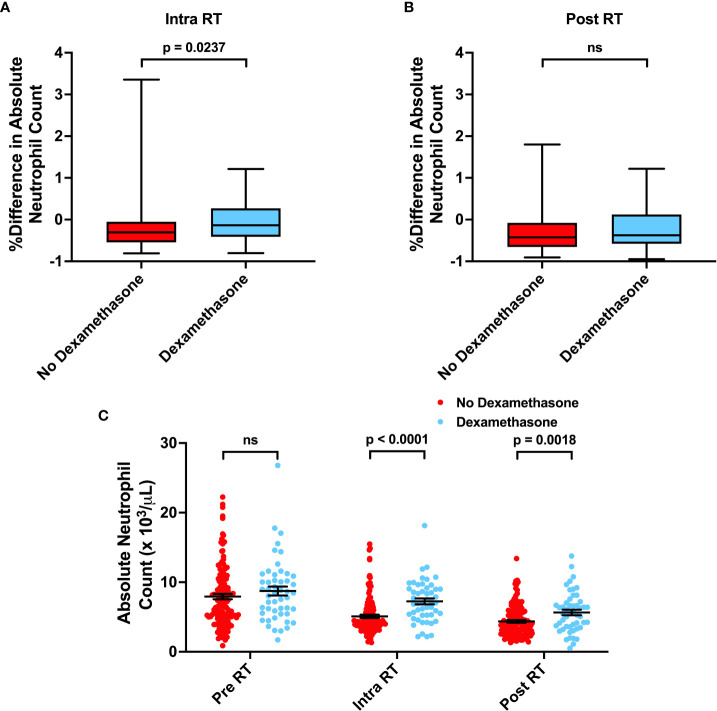
Associations between Dexamethasone use and Neutrophil Counts. **(A, B)** Percent difference in neutrophil counts for patients not receiving or receiving dexamethasone concurrently with radiation. **(C)** Absolute neutrophil counts before, during, and after RT for patients not receiving or receiving dexamethasone concurrently with radiation. Statistical analysis was performed using two sample t-tests. ns, not significant.

Neutrophils have been associated with increased resistance to radiation and poor cancer patient outcomes. Furthermore, elevated neutrophil-to-lymphocyte ratios (NLR > 4.0) have also been observed to be a poor prognostic indicator in glioma patients (8–10). Thus, we evaluated the effects of neutrophils and neutrophilia on survival. Neutrophilia in the time intervals before (Adj. Hazard Ratio 1.706, 95% CI 1.094 to 2.662), during (Adj. Hazard Ratio 1.641, 95% CI 1.056 to 2.550), and after (Adj. Hazard Ratio 1.517, 95% CI 0.940 to 2.449) radiation therapy were all associated with poorer OS. Similarly, neutrophilia during radiation (Adj. Hazard Ratio 1.617, 95% CI 1.057 to 2.474) was associated with poorer PFS (**Table 2**; **Figure 2** and **Supplementary Table 4**). Pre-radiation NLR > 4.0 was associated with worse OS (Adj. Hazard Ratio 1.847, 95% CI 1.218 to 2.803) and trended towards worse PFS (Adj. Hazard Ratio 1.430, 95% CI 0.968 to 2.112) (**Table 2**; **Figure 3**). Lastly, as with the case with lymphocytes, patients who received higher fractions of radiation also exhibited a percent decrease and lower absolute count of neutrophils, but both during and after radiation treatment (**Figure 4**). Patients who used dexamethasone during radiation therapy exhibited elevated neutrophil levels during and after radiation treatment (**Figure 5**). Concurrent temozolomide use was not associated with significant changes in neutrophil counts (**Supplementary Figures 3C, D**). Overall, our results observe worse survival outcomes with higher neutrophil counts.

**Table 2:** Multivariable cox proportional hazards regression: Impact of neutrophils on OS and PFS.

	OS			PFS		
Variable	Hazard ratio (Adj.)	95% CI	p value	Hazard ratio (Adj.)	95% CI	p value
Pre-RT Neutrophilia	1.706	1.094 - 2.662	0.0185	1.471	0.967 - 2.238	0.0712
Intra-RT Neutrophilia	1.641	1.056 - 2.550	0.0277	1.617	1.057 - 2.474	0.0267
Post-RT Neutrophilia	1.517	0.940 - 2.449	0.0879	1.382	0.864 - 2.210	0.1765
Pre-RT NLR > 4	1.847	1.218 - 2.803	0.0039	1.430	0.968 - 2.112	0.0724
Intra-RT NLR > 4	1.549	1.036 - 2.316	0.0331	1.311	0.888 - 1.935	0.1736
Post-RT NLR > 4	1.462	0.964 - 2.217	0.0740	1.167	0.792 - 1.718	0.4354
Intra-RT %Δ Neutrophils	1.254	0.717 - 2.193	0.4273	1.372	0.820 - 2.297	0.2281
Post-RT %Δ Neutrophils	0.909	0.562 - 1.471	0.6984	0.930	0.593 - 1.460	0.7537

OS, Overall Survival; PFS, Progression Free Survival; RT, Radiation therapy; NLR, Neutrophil to lymphocyte ratio; CI, Confidence Interval.

Hazard ratios for neutrophilia and NLR were calculated as categorical variables, while percent changes were calculated as continuous variables. Variables were adjusted for number of radiation fractions, age, BMI, gender, p53 mutation status, MGMT methylation status, presence of edema, presence of seizures, concurrent temozolomide treatment, and concurrent dexamethasone treatment.

**Figure 6:**
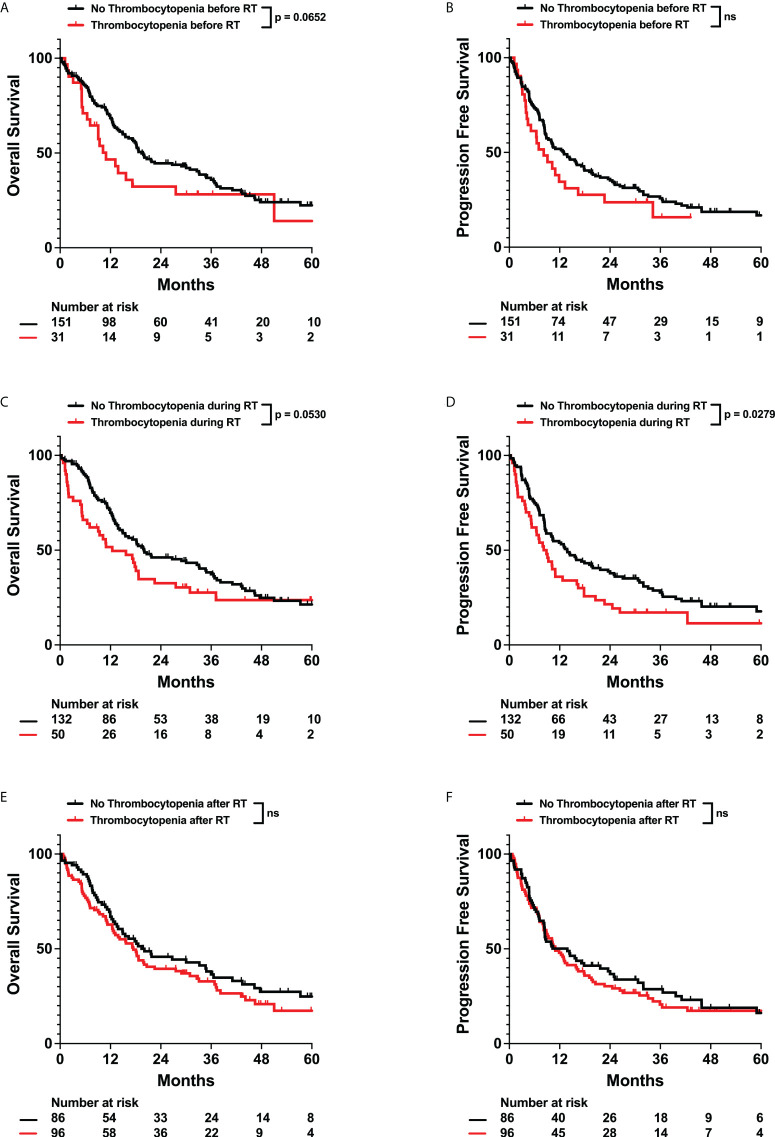
Effects of Thrombocytopenia on OS and PFS. Kaplan-Meyer plots of OS and PFS comparing patients with thrombocytopenia **(A, B)** before, **(C, D)** during, **(E, F)** or after radiation treatment. Statistical analysis was performed using Cox Proportional Hazards tests. OS, Overall Survival; PFS, Progression Free Survival; RT, Radiation therapy. “ns” for all figures stands for not significant.

**Figure 7:**
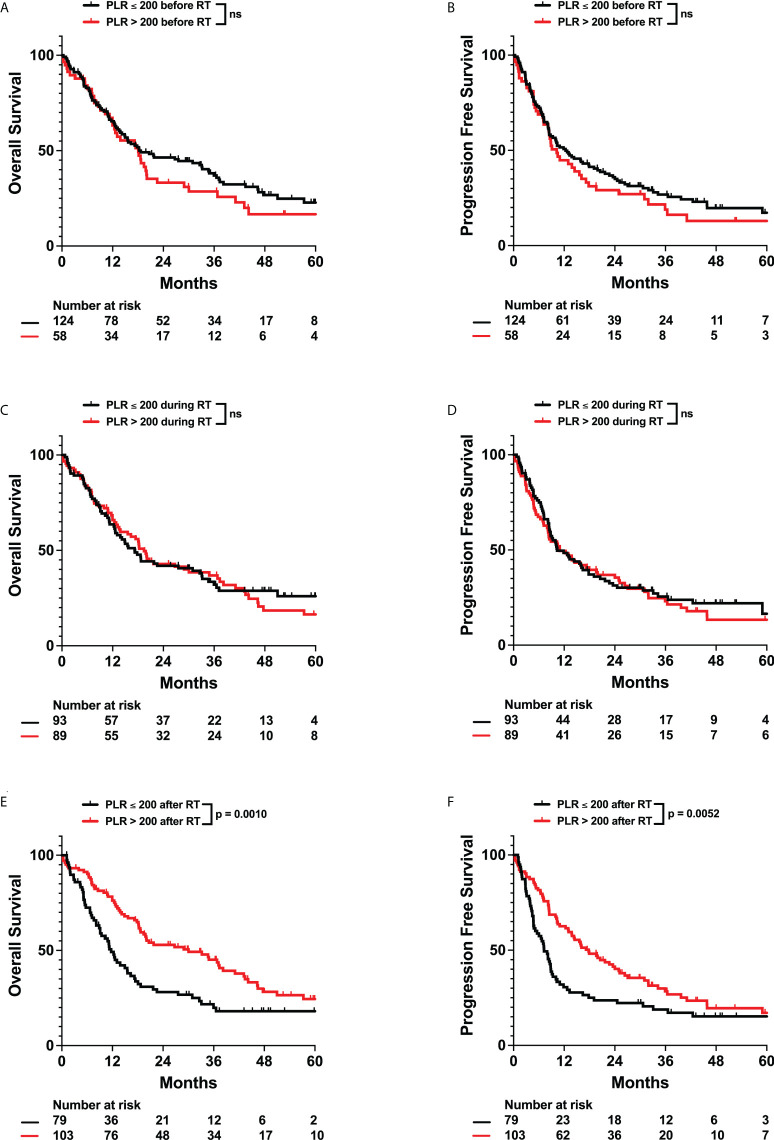
Effects of Platelet to Lymphocyte Ratio (PLR) on OS and PFS. Kaplan-Meyer plots of OS and PFS comparing patients with PLR > 200 **(A, B)** before, **(C, D)** during, **(E, F)** or after radiation treatment. Statistical analysis was performed using Cox Proportional Hazards tests. OS, Overall Survival; PFS, Progression Free Survival; PLR, Platelet to Lymphocyte Ratio; RT, Radiation therapy. ns, not significant.

**Figure 8:**
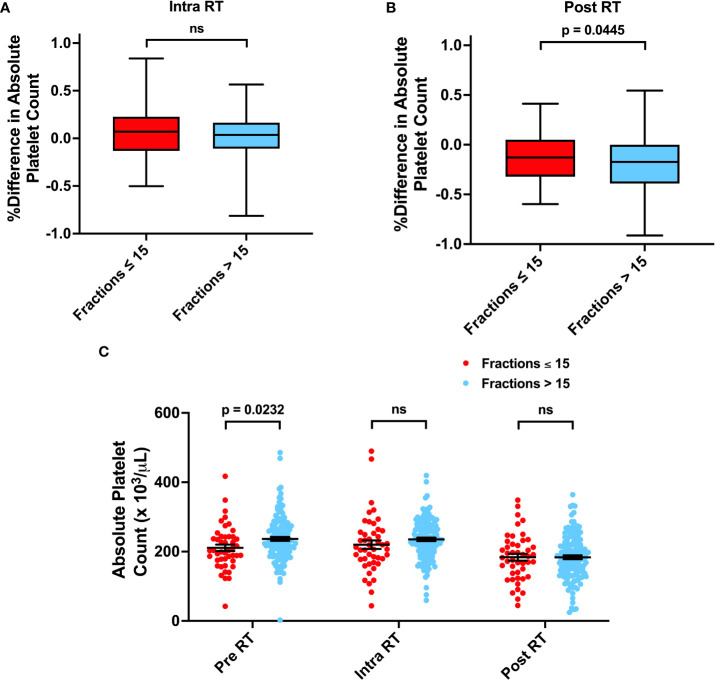
Associations between Number of Radiation Fractions and Platelet Counts. **(A, B)** Percent difference in platelet counts for patients receiving ≤ 15 or > 15 fractions from before radiation treatment (RT) to during RT or after RT. **(C)** Absolute platelet counts before, during, and after RT for patients receiving ≤ 15 or > 15 fractions. Statistical analysis was performed using two sample t-tests. ns, not significant.

Previous studies have observed that high pretreatment platelet-to-lymphocyte ratios (PLR > 200) have been correlated with poor prognosis in gliomas. However, the relationship between overall platelet counts and survival in solid tumor patients is unclear. We did not observe any significant impact of pre-radiation thrombocytopenia or elevated PLR on survival. However, we observed that thrombocytopenia in the intra-radiation time interval was associated with worse OS (Adj. Hazard Ratio 1.531, 95% CI 1.009 to 2.323) (**Figure 6**; **Table 3**, and **Supplementary Table 5**). Post-radiation PLR > 200 was significantly associated with improved OS (Adj. Hazard Ratio 0.587, 95% CI 0.401 to 0.860) and PFS (Adj. Hazard Ratio 0.681, 95% CI 0.477 to 0.974) (**Table 3**; **Figure 7**). Consistently, a percent increase in platelet counts after radiation was also significantly associated with improved OS (Adj. Hazard Ratio 0.387, 95% CI 0.192 to 0.778) and PFS (Adj. Hazard Ratio 0.491, 95% CI 0.252 to 0.956) (**Table 3**; **Supplementary Table 5**). Lastly, patients who received more than 15 fractions of radiation exhibited significant a percent decrease in platelet counts after radiation treatment (**Figure 8**). Temozolomide nor dexamethasone use was not associated with changes in platelet counts (**Supplementary Figures 3E, F, 4C, D**). Overall, these results correlate a post-radiation thrombocytopenic state with worse survival outcomes.

**Table 3:** Multivariable cox proportional hazards regression: Impact of platelets on OS and PFS.

	OS			PFS		
Variable	Hazard ratio (Adj.)	95% CI	p value	Hazard ratio (Adj.)	95% CI	p value
Pre-RT Thrombocytopenia	1.059	0.654 - 1.714	0.8165	0.956	0.598 - 1.528	0.8496
Intra-RT Thrombocytopenia	1.531	1.009 - 2.323	0.0455	1.403	0.943 - 2.087	0.0949
Post-RT Thrombocytopenia	1.377	0.939 - 2.019	0.1019	1.158	0.810 - 1.655	0.4215
Pre-RT PLR > 200	1.000	0.662 - 1.512	0.9991	1.000	0.674 - 1.484	0.9997
Intra-RT PLR > 200	1.025	0.698 - 1.505	0.8988	1.151	0.795 - 1.666	0.4562
Post-RT PLR > 200	0.587	0.401 - 0.860	0.0062	0.681	0.477 - 0.974	0.0352
Intra-RT %Δ Platelets	0.500	0.227 - 1.101	0.0854	0.598	0.280 - 1.278	0.1846
Post-RT %Δ Platelets	0.387	0.192 - 0.778	0.0077	0.491	0.252 - 0.956	0.0365

OS. Overall Survival; PFS, Progression Free Survival; RTm Radiation therapy; PLR, Platelet to lymphocyte ratio; CI, Confidence Interval.

Hazard ratios for thrombocytopenia and PLR were calculated as categorical variables, while percent changes were calculated as continuous variables. Variables were adjusted for number of radiation fractions, age, BMI, gender, p53 mutation status, MGMT methylation status, presence of edema, presence of seizures, concurrent temozolomide treatment, and concurrent dexamethasone treatment.

Monocytes have also been observed to correlate with poor prognosis in the form of elevated monocyte to lymphocyte ratio (MLR) or decreased lymphocyte to monocyte (LMR) ratios. Thus, we evaluated whether monocytosis, MLR > 0.5, or changes in monocytes had significant impacts on survival. We did not observe any significant correlations between monocytosis, MLR, or percent change in monocyte counts at any time interval with survival (**Supplementary Tables 6, 7**). Furthermore, number of radiation fractions, temozolomide use, and dexamethasone use did not appear to be related to changes in absolute monocyte counts (**Supplementary Figures 3G, H, 4E, F, 5**).

## Discussion

Gliomas are the most prevalent adult brain tumor, and despite advances in therapy, glioblastomas are nearly universally fatal. Therefore, it is vital to determine which factors, including hematologic state, accurately contribute to poor survival. It is unclear how treatment induced changes in cell counts, especially those due to radiation, chemotherapy, and steroid use, contribute to survival. Therefore, we assessed how lymphocyte, neutrophil, platelet, and monocyte levels before, during, and after radiation therapy correlated with patient survival. To our knowledge, this is the first study that has evaluated the impact of not only pre-treatment hematologic state, but also intra- and post-radiation hematologic states in glioma patients.

Our cohort of 182 patients exhibited a median OS and PFS of 18.2 and 11.0 months, respectively, which is consistent with other studies (**Figure 1**) (21, 22). From the noted patient characteristics, a higher number of radiation fractions, lower age, positive IDH mutant status, and lack of dexamethasone use during radiation were significantly associated with improved survival (**Supplementary Table 1**) (23–25). For patients with high-grade gliomas, we typically prescribe a dose of 59.4 Gy in 33 fractions or 60 Gy in 30 fractions, while low-grade gliomas are typically treated to a total dose of 45 to 54 Gy in 1.8-2.0 Gy fractions (7, 26–31). However, in some patients with poor performance status, especially those with glioblastomas, we typically prescribe a short-course radiation schedule of 40 Gy in 15 fractions or 25 Gy in 5 fractions (32–34). In many patients receiving short-course radiation therapy, we also include temozolomide, which has been previously demonstrated to confer a survival benefit (35). Dexamethasone can also be used in patients who may exhibit cerebral edema. With the strong correlation between radiation fractions and patient outcome, our primary question investigated whether various hematologic states could predict for survival independently from radiation fraction number.

Lymphopenia has been observed to correlate with poor patient outcomes (9, 36). In our study, contrary to previous studies, we initially observed a significant univariate correlation between post-radiation lymphopenia and improved survival (**Supplementary Figure 1**, **Supplementary Table 2**). However, after adjusting for patient characteristics, including number of radiation fractions, this association was abolished (**Supplementary Table 3**). This adjustment suggests that number of radiation fractions may be a confounding factor when considering post-radiation lymphopenic changes as a prognostic marker, which is consistent with our observation of decreased lymphocyte counts for patients receiving higher radiation fractions (**Supplementary Figure 2**).

Neutrophils have been observed to promote cancers in numerous ways, for example through host inflammation, which is a major hallmark of cancer (37). They can also induce tumor initiation, growth, proliferation, and metastasis (38, 39). Therefore, multiple studies have turned to studying neutrophil levels and systemic inflammation marker NLR as prognostic predictors of outcome (40). In glioma patients, preoperative NLR > 4.0 has previously been correlated with worse survival (8, 10, 41). Consistently with these studies, our data also identifies pre-radiation NLR > 4.0 as an independent predictor of worse OS and PFS (**Table 2**; **Figure 3**). However, as neutrophils have been observed to promote resistance to radiation, we also evaluated neutrophil levels during and after radiation treatment (42). We observed that neutrophilia both during and after radiation were associated with worse survival (**Figure 2**, **Table 3**). This was significant even after adjusting for radiation fraction number, which is correlated with a decrease in neutrophil levels, and dexamethasone use, which is associated with an increase in neutrophil levels (**Figures 4**, **5**). As such, worse outcomes in patients with post-radiation neutrophilia cannot be attributed to insufficient radiation or steroid use during radiation. Thus, our results suggest that neutrophilia at any time interval surrounding radiation treatment serves as an independent predictor of poor survival, even after factoring in the significant impact of treatments on neutrophil counts. Determining which patients have high neutrophil counts can guide further intensification of treatment with additional systemic therapy, which may improve prognosis. For example, multiple clinical trials (NCT01220271, NCT01582269) are evaluating the efficacy of adjuvant TGF-beta inhibitor Galunisertib in malignant gliomas in combination with chemotherapy or chemoradiation. One potential mechanism of action of Galunisertib is to inhibit TGF-beta induced polarization of neutrophils into a protumor phenotype (43, 44). Such adjuvant treatments may thus be especially effective in patients observed to exhibit neutrophilia.

The role of platelets as a prognostic factor in solid cancer patients is unclear. Some studies have observed that high platelet counts correlate with poor outcome – attributing this observation to platelet secreted cytokines that facilitate tumor growth and angiogenesis or an inflammatory state in the form of elevated preoperative PLR (8, 10, 45–47). On the contrary, other groups have argued that thrombocytopenia contributes to poor survival. Numerous factors that contribute to thrombocytopenia, such as genetic abnormalities in the tumor, treatment side effects, or immune cell imbalance leading to platelet destruction, can all portend poor prognosis (48). In our patient cohort, after adjusting for patient characteristics including radiation fraction number, we observe that patients with post-radiation increases in platelet counts or PLR > 200 exhibited improved survival (**Table 3**, **Figure 7**). This is independent of increased radiation dose, as increased radiation fractions are associated with decreased platelet counts (**Figure 8**). To our knowledge, no previous studies have evaluated or observed an association between post-treatment thrombocytopenia with worse survival in glioma patients. Therefore, the mechanisms of how low platelet counts impact patient survival must be further elucidated.

One other inflammatory marker that has been suggested to be correlated with poor survival in multiple different malignancies is MLR or its inverse LMR (49). Monocytes can influence tumor progression by stimulating angiogenesis and metastasis, influencing antitumor immunity, and differentiating into tumor-associate macrophages (50). Low preoperative LMR has been observed to be an independent prognostic factor in gastric cancer patients, and a small retrospective study indicates that glioblastoma patients with elevated MLR exhibit shorter postoperative survival (51, 52). However, our results ultimately did not find any correlation between monocytosis, MLR, or changes in monocyte counts with survival (**Supplementary Table 7**). Further larger scale and mechanistic studies may be required to further delineate the role of monocytes in patient prognosis.

Our study had the traditional limitations that are relevant to all retrospective evaluations. These weaknesses include non-random treatment group allocation, selection bias, and non-random loss to follow up intrinsic to any non-randomized non-prospective study (53). Despite this, our study still accounts for loss to follow up during statistical analysis. Furthermore, the number and time of collection of patient blood samples for hematologic counts were not equivalent throughout our patient population. Lastly, alternative confounders that may affect hematologic cell counts, such as genetic alterations to the tumor, were not incorporated into our analysis.

Taken together, our study provides insight into hematologic predictors of survival in glioma patients. Consistent with previous studies, we observe that elevated pre-radiation NLR predicts for poor prognosis. In addition to this, we have identified that neutrophilia at any time interval surrounding radiotherapy and post-radiation thrombocytopenia are also correlated with worse patient prognosis. We found no significant correlation between lymphocyte counts or monocyte counts and patient survival. These results can inform clinicians on when potential adjuvant therapies involving neutrophils or platelets may provide further benefit to patient survival.

## Author's note

The content is solely the responsibility of the authors and does not necessarily represent the official views of the NIH.

## Data availability statement

The raw data supporting the conclusions of this article will be made available by the authors, without undue reservation.

## Ethics statement

The studies involving human participants were reviewed and approved by UT Southwestern Institutional Review Board. Written informed consent for participation was not required for this study in accordance with the national legislation and the institutional requirements.

## Author contributions

Conceptualization: EH, TD, DV. Methodology: EH, JT, DV. Formal Analysis: EH, ML. Investigation: EH, JT, EM, MY, RT, ZW, TP, TD, DV. Original Draft: EH, DV. Draft Review: EH, JT, EM, MY, RT, ZW, TP, TD, DV. Supervision: DV. All authors contributed to the article and approved the submitted version.

## Acknowledgments

We thank the UT Southwestern Radiation Oncology and Neurological Surgery Departments for their support and organization of patient care. Specifically, we would like to thank Drs. Wen Jiang and Lucien Nedzi for their involvement in clinical care, follow-up of patients, and scientific discussion. Study data were collected and managed using the Clinical Data Exchange Network (ClinDEN) hosted by UT Southwestern Medical Center and supported by CTSA Grant Number UL1 TR003163 from the National Center for Advancing Translational Science (NCATS), a component of the National Institutes of Health (NIH).

## Conflict of interest

Author RT is on the board of directors for TRIO corporation, TMIT corporation, and Reflexion. All do not relate to the subject of this study. Author DV has research funding from AstraZeneca. All do not relate to the subject of this study.

The remaining authors declare that the research was conducted in the absence of any commercial or financial relationships that could be construed as a potential conflict of interest.

## Publisher’s note

All claims expressed in this article are solely those of the authors and do not necessarily represent those of their affiliated organizations, or those of the publisher, the editors and the reviewers. Any product that may be evaluated in this article, or claim that may be made by its manufacturer, is not guaranteed or endorsed by the publisher.
