# Supplementary material for: Neutrophilia and post-radiation thrombocytopenia predict for poor prognosis in radiation-treated glioma patients
Source: Front Oncol. 2022 Sep 9;12:1000280. doi: 10.3389/fonc.2022.1000280 (PMC9501690; doi:10.3389/fonc.2022.1000280)
Supplement: Supplementary file 1 [file DataSheet_1.docx]

**Supplementary Figures and Tables**

**Supplementary Table 1.** Univariate Cox Proportional Hazards Regression: Impact of Patient Characteristics on OS and PFS. Hazard ratios for number of fractions, age, and BMI were calculated as continuous variables, while all other variables were calculated as categorical variables. Abbreviations: OS = Overall Survival, PFS = Progression Free Survival, BMI = Body Mass Index, IDH = Isocitrate Dehydrogenase, MGMT = 06-Methylguanine-DNA Methyltransferase, Temo = Temozolomide, Dexa = Dexamethasone, CI = Confidence Interval.

**Supplementary Figure 1.** Effects of Lymphopenia on OS and PFS. Kaplan-Meyer plots of OS and PFS comparing patients with lymphopenia **(A-B)** before, **(C-D)** during, **(E-F)** or after radiation treatment. Statistical analysis was performed using Cox Proportional Hazards tests. Abbreviations: OS = Overall Survival, PFS = Progression Free Survival, RT = Radiation therapy.

**Supplementary Table 2.** Univariate Cox Proportional Hazards Regression: Impact of lymphocytes on OS and PFS. Hazard ratios for lymphopenia were calculated as categorical variables, while percent changes were calculated as continuous variables. Abbreviations: OS = Overall Survival, PFS = Progression Free Survival, RT = Radiation therapy, CI = Confidence Interval.

**Supplementary Table 3.** Multivariable Cox Proportional Hazards Regression: Impact of lymphocytes on OS and PFS. Hazard ratios for lymphopenia were calculated as categorical variables, while percent changes were calculated as continuous variables. Variables were adjusted for number of radiation fractions, age, BMI, gender, p53 mutation status, MGMT methylation status, presence of edema, presence of seizures, concurrent temozolomide treatment, and concurrent dexamethasone treatment. Abbreviations: OS = Overall Survival, PFS = Progression Free Survival, RT = Radiation therapy, CI = Confidence Interval.

**Supplementary Figure 2.** Associations between Number of Radiation Fractions and Lymphocyte Counts. **(A-B)** Percent difference in lymphocyte counts for patients receiving ≤ 15 or > 15 fractions from before radiation treatment (RT) to during RT or after RT. **(C)** Absolute lymphocyte counts before, during, and after RT for patients receiving ≤ 15 or > 15 fractions. Statistical analysis was performed using two sample t-tests.

**Supplementary Figure 3.** Associations between Temozolomide use and Cell Counts. Percent difference in **(A-B)** lymphocyte, **(C-D)** platelet, or **(E-F)** monocyte counts for patients either not receiving or receiving dexamethasone therapy concurrently with RT. Statistical analysis was performed using two sample t-tests.

**Supplementary Figure 4.** Associations between Dexamethasone use and Cell Counts. Percent difference in **(A-B)** lymphocyte, **(C-D)** neutrophil, **(E-F)** platelet, or **(G-H)** monocyte counts for patients either not receiving or receiving temozolomide therapy concurrently with RT. Statistical analysis was performed using two sample t-tests.

**Supplementary Table 4.** Univariate Cox Proportional Hazards Regression: Impact of neutrophils on OS and PFS. Hazard ratios for neutrophilia and NLR were calculated as categorical variables, while percent changes were calculated as continuous variables. Abbreviations: OS = Overall Survival, PFS = Progression Free Survival, RT = Radiation therapy, NLR = Neutrophil to lymphocyte ratio, CI = Confidence Interval.

**Supplementary Table 5.** Univariate Cox Proportional Hazards Regression: Impact of platelets on OS and PFS. Hazard ratios for thrombocytopenia and PLR were calculated as categorical variables, while percent changes were calculated as continuous variables. Abbreviations: OS = Overall Survival, PFS = Progression Free Survival, RT = Radiation therapy, PLR = Platelet to lymphocyte ratio, CI = Confidence Interval.

**Supplementary Table 6.** Univariate Cox Proportional Hazards Regression: Impact of monocytes on OS and PFS. Hazard ratios for monocytosis and MLR were calculated as categorical variables, while percent changes were calculated as continuous variables. Abbreviations: OS = Overall Survival, PFS = Progression Free Survival, RT = Radiation therapy, MLR = Monocyte to lymphocyte ratio, CI = Confidence Interval.

**Supplementary Table 7.** Multivariable Cox Proportional Hazards Regression: Impact of monocytes on OS and PFS. Hazard ratios for monocytosis and MLR were calculated as categorical variables, while percent changes were calculated as continuous variables. Variables were adjusted for number of radiation fractions, age, BMI, gender, p53 mutation status, MGMT methylation status, presence of edema, presence of seizures, concurrent temozolomide treatment, and concurrent dexamethasone treatment. Abbreviations: OS = Overall Survival, PFS = Progression Free Survival, RT = Radiation therapy, MLR = Monocyte to lymphocyte ratio, CI = Confidence Interval.

**Supplementary Figure 5.** Associations between Number of Radiation Fractions and Monocyte Counts. **(A-B)** Percent difference in monocyte counts for patients receiving ≤ 15 or > 15 fractions from before radiation treatment (RT) to during RT or after RT. **(C)** Absolute monocyte counts before, during, and after RT for patients receiving ≤ 15 or > 15 fractions. Statistical analysis was performed using two sample t-tests.

**Supplementary Table 1.** Impact of Patient Characteristics on OS and PFS.

|  | **OS** | | | **PFS** | | |
| --- | --- | --- | --- | --- | --- | --- |
| **Variable** | **Hazard Ratio** | **95% CI** | **p value** | **Hazard Ratio** | **95% CI** | **p value** |
| **Fractions** | 0.924 | 0.902 - 0.947 | 1.8E-10 | 0.931 | 0.909 - 0.953 | 3.2E-09 |
|  |  |  |  |  |  |  |
| **Age** | 1.038 | 1.024 - 1.052 | 1.0E-07 | 1.030 | 1.017 - 1.043 | 7.0E-06 |
|  |  |  |  |  |  |  |
| **BMI** | 1.002 | 0.971 - 1.035 | 0.8874 | 1.002 | 0.972 - 1.034 | 0.8845 |
|  |  |  |  |  |  |  |
| **Gender** | 1.223 | 0.856 - 1.747 | 0.2684 | 1.215 | 0.860 - 1.717 | 0.2698 |
|  |  |  |  |  |  |  |
| **IDH Mutation** | 0.166 | 0.089 - 0.310 | 1.7E-08 | 0.166 | 0.093 - 0.296 | 1.2E-09 |
|  |  |  |  |  |  |  |
| **P53 Mutation** | 0.531 | 0.285 - 0.991 | 0.0469 | 0.514 | 0.283 - 0.935 | 0.0291 |
|  |  |  |  |  |  |  |
| **MGMT Methylation** | 0.609 | 0.327 - 1.131 | 0.1164 | 0.611 | 0.345 - 1.084 | 0.0922 |
|  |  |  |  |  |  |  |
| **Presence of Edema** | 1.428 | 1.012 - 2.017 | 0.0428 | 1.356 | 0.970 - 1.896 | 0.0747 |
|  |  |  |  |  |  |  |
| **Presence of Seizures** | 0.572 | 0.394 - 0.830 | 0.0033 | 0.655 | 0.459 - 0.934 | 0.0196 |
|  |  |  |  |  |  |  |
| **Concurrent Temo** | 0.797 | 0.531 - 1.198 | 0.2749 | 0.897 | 0.642 - 1.253 | 0.5220 |
|  |  |  |  |  |  |  |
| **Concurrent Dexa** | 1.689 | 1.148 - 2.484 | 0.0078 | 1.652 | 1.147 - 2.379 | 0.0070 |
|  |  |  |  |  |  |  |

**Supplementary Figure 1.** Effects of Lymphopenia on OS and PFS.

**Supplementary Table 2.** Univariate analysis of impact of lymphocytes on OS and PFS.

|  | **OS** | | | **PFS** | | |
| --- | --- | --- | --- | --- | --- | --- |
| **Variable** | **Hazard Ratio** | **95% CI** | **p value** | **Hazard Ratio** | **95% CI** | **p value** |
| **Pre-RT Lymphopenia** | 1.468 | 1.030 - 2.092 | 0.0336 | 1.480 | 1.052 - 2.081 | 0.0244 |
|  |  |  |  |  |  |  |
| **Intra-RT Lymphopenia** | 1.090 | 0.719 - 1.651 | 0.6859 | 1.278 | 0.856 - 1.908 | 0.2307 |
|  |  |  |  |  |  |  |
| **Post-RT Lymphopenia** | 0.543 | 0.323 - 0.913 | 0.0212 | 0.652 | 0.390 - 1.088 | 0.1018 |
|  |  |  |  |  |  |  |
| **Intra-RT %Δ Lymphocytes** | 1.216 | 0.854 - 1.733 | 0.2780 | 1.154 | 0.814 - 1.636 | 0.4220 |
|  |  |  |  |  |  |  |
| **Post-RT %Δ Lymphocytes** | 1.965 | 1.322 - 2.919 | 0.0008 | 1.866 | 1.285 - 2.711 | 0.0011 |
|  |  |  |  |  |  |  |

**Supplementary Table 3.** Multivariable analysis of impact of lymphocytes on OS and PFS.

|  | **OS** | | | **PFS** | | |
| --- | --- | --- | --- | --- | --- | --- |
| **Variable** | **Hazard Ratio (Adj.)** | **95% CI** | **p value** | **Hazard Ratio (Adj.)** | **95% CI** | **p value** |
| **Pre-RT Lymphopenia** | 1.029 | 0.677 - 1.566 | 0.8922 | 1.145 | 0.767 - 1.710 | 0.5086 |
|  |  |  |  |  |  |  |
| **Intra-RT Lymphopenia** | 1.332 | 0.835 - 2.127 | 0.2289 | 1.644 | 1.037 - 2.606 | 0.0345 |
|  |  |  |  |  |  |  |
| **Post-RT Lymphopenia** | 0.884 | 0.480 - 1.627 | 0.6921 | 1.076 | 0.598 - 1.937 | 0.8076 |
|  |  |  |  |  |  |  |
| **Intra-RT %Δ Lymphocytes** | 1.203 | 0.773 - 1.872 | 0.4130 | 1.071 | 0.679 - 1.691 | 0.7682 |
|  |  |  |  |  |  |  |
| **Post-RT %Δ Lymphocytes** | 1.221 | 0.716 - 2.082 | 0.4631 | 1.140 | 0.675 - 1.924 | 0.6244 |
|  |  |  |  |  |  |  |

**Supplementary Figure 2.** Associations between Number of Radiation Fractions and Lymphocyte Counts.

**Supplementary Figure 3.** Associations between Temozolomide use and Cell Counts

**Supplementary Figure 4.** Associations between Dexamethasone use and Cell Counts

**Supplementary Table 4.** Univariate analysis of impact of neutrophils on OS and PFS.

|  | **OS** | | | **PFS** | | |
| --- | --- | --- | --- | --- | --- | --- |
| **Variable** | **Hazard Ratio** | **95% CI** | **p value** | **Hazard Ratio** | **95% CI** | **p value** |
| **Pre-RT Neutrophilia** | 1.513 | 1.038 - 2.206 | 0.0313 | 1.365 | 0.959 - 1.942 | 0.0844 |
|  |  |  |  |  |  |  |
| **Intra-RT Neutrophilia** | 1.830 | 1.280 - 2.616 | 0.0009 | 1.799 | 1.280 - 2.528 | 0.0007 |
|  |  |  |  |  |  |  |
| **Post-RT Neutrophilia** | 2.260 | 1.567 - 3.261 | 1.3E-5 | 2.082 | 1.464 - 2.961 | 4.5E-5 |
|  |  |  |  |  |  |  |
| **Pre-RT NLR > 4** | 2.001 | 1.336 - 2.998 | 0.0008 | 1.682 | 1.159 - 2.442 | 0.0062 |
|  |  |  |  |  |  |  |
| **Intra-RT NLR > 4** | 1.419 | 0.992 - 2.030 | 0.0556 | 1.391 | 0.986 - 1.962 | 0.0599 |
|  |  |  |  |  |  |  |
| **Post-RT NLR > 4** | 1.225 | 0.834 - 1.799 | 0.2999 | 1.111 | 0.773 - 1.598 | 0.5684 |
|  |  |  |  |  |  |  |
| **Intra-RT %Δ Neutrophils** | 1.808 | 1.198 - 2.731 | 0.0048 | 1.961 | 1.339 - 2.872 | 0.0005 |
|  |  |  |  |  |  |  |
| **Post-RT %Δ Neutrophils** | 1.133 | 0.789 - 1.626 | 0.4997 | 1.154 | 0.823 - 1.617 | 0.4059 |
|  |  |  |  |  |  |  |

**Supplementary Table 5.** Univariate analysis of impact of platelets on OS and PFS.

|  | **OS** | | | **PFS** | | |
| --- | --- | --- | --- | --- | --- | --- |
| **Variable** | **Hazard Ratio** | **95% CI** | **p value** | **Hazard Ratio** | **95% CI** | **p value** |
| **Pre-RT Thrombocytopenia** | 1.534 | 0.973 - 2.417 | 0.0652 | 1.362 | 0.874 - 2.123 | 0.1719 |
|  |  |  |  |  |  |  |
| **Intra-RT Thrombocytopenia** | 1.462 | 0.995 - 2.149 | 0.0530 | 1.505 | 1.045 - 2.167 | 0.0279 |
|  |  |  |  |  |  |  |
| **Post-RT Thrombocytopenia** | 1.268 | 0.890 - 1.806 | 0.1885 | 1.102 | 0.788 - 1.542 | 0.5700 |
|  |  |  |  |  |  |  |
| **Pre-RT PLR > 200** | 1.175 | 0.808 - 1.707 | 0.3983 | 1.187 | 0.831 - 1.696 | 0.3470 |
|  |  |  |  |  |  |  |
| **Intra-RT PLR > 200** | 1.030 | 0.726 - 1.461 | 0.8688 | 1.106 | 0.792 - 1.544 | 0.5545 |
|  |  |  |  |  |  |  |
| **Post-RT PLR > 200** | 0.550 | 0.386 - 0.784 | 0.0010 | 0.618 | 0.441 - 0.866 | 0.0052 |
|  |  |  |  |  |  |  |
| **Intra-RT %Δ Platelets** | 0.956 | 0.737 - 1.239 | 0.7313 | 0.940 | 0.607 - 1.456 | 0.7831 |
|  |  |  |  |  |  |  |
| **Post-RT %Δ Platelets** | 0.743 | 0.390 - 1.413 | 0.3650 | 0.815 | 0.440 - 1.509 | 0.5154 |
|  |  |  |  |  |  |  |

**Supplementary Table 6.** Univariate analysis of impact of monocytes on OS and PFS.

|  | **OS** | | | **PFS** | | |
| --- | --- | --- | --- | --- | --- | --- |
| **Variable** | **Hazard Ratio** | **95% CI** | **p value** | **Hazard Ratio** | **95% CI** | **p value** |
| **Pre-RT Monocytosis** | 1.064 | 0.887 - 1.277 | 0.5031 | 1.026 | 0.864 - 1.218 | 0.7687 |
|  |  |  |  |  |  |  |
| **Intra-RT Monocytosis** | 0.904 | 0.750 - 1.090 | 0.2910 | 0.921 | 0.781 - 1.086 | 0.3261 |
|  |  |  |  |  |  |  |
| **Post-RT Monocytosis** | 1.218 | 0.974 - 1.523 | 0.0844 | 1.216 | 0.989 - 1.495 | 0.0641 |
|  |  |  |  |  |  |  |
| **Pre-RT MLR > 0.5** | 1.059 | 0.729 - 1.537 | 0.7651 | 1.207 | 0.862 - 1.691 | 0.2738 |
|  |  |  |  |  |  |  |
| **Intra-RT MLR > 0.5** | 1.012 | 0.702 - 1.459 | 0.9500 | 1.106 | 0.792 - 1.545 | 0.5547 |
|  |  |  |  |  |  |  |
| **Post-RT MLR > 0.5** | 0.754 | 0.530 - 1.071 | 0.1151 | 0.811 | 0.573 - 1.150 | 0.2403 |
|  |  |  |  |  |  |  |
| **Intra-RT %Δ Monocytes** | 1.015 | 0.666 - 1.548 | 0.9434 | 1.370 | 0.898 - 2.090 | 0.1436 |
|  |  |  |  |  |  |  |
| **Post-RT %Δ Monocytes** | 0.993 | 0.599 - 1.647 | 0.9787 | 1.453 | 0.871 - 2.424 | 0.1520 |
|  |  |  |  |  |  |  |

**Supplementary Table 7.** Multivariable analysis of impact of monocytes on OS and PFS.

|  | **OS** | | | **PFS** | | |
| --- | --- | --- | --- | --- | --- | --- |
| **Variable** | **Hazard Ratio (Adj.)** | **95% CI** | **p value** | **Hazard Ratio (Adj.)** | **95% CI** | **p value** |
| **Pre-RT Monocytosis** | 0.921 | 0.754 - 1.125 | 0.4220 | 0.874 | 0.721 - 1.060 | 0.1701 |
|  |  |  |  |  |  |  |
| **Intra-RT Monocytosis** | 0.834 | 0.662 - 1.050 | 0.1228 | 0.819 | 0.665 - 1.009 | 0.0609 |
|  |  |  |  |  |  |  |
| **Post-RT Monocytosis** | 1.060 | 0.808 - 1.391 | 0.6749 | 1.016 | 0.787 - 1.311 | 0.9057 |
|  |  |  |  |  |  |  |
| **Pre-RT MLR > 0.5** | 1.085 | 0.743 - 1.586 | 0.6724 | 0.910 | 0.630 - 1.315 | 0.6145 |
|  |  |  |  |  |  |  |
| **Intra-RT MLR > 0.5** | 1.155 | 0.796 - 1.674 | 0.4483 | 1.034 | 0.724 - 1.475 | 0.8546 |
|  |  |  |  |  |  |  |
| **Post-RT MLR > 0.5** | 0.972 | 0.654 - 1.445 | 0.8891 | 0.907 | 0.621 - 1.324 | 0.6125 |
|  |  |  |  |  |  |  |
| **Intra-RT %Δ Monocytes** | 1.015 | 0.621 - 1.657 | 0.9536 | 1.344 | 0.819 - 2.204 | 0.2422 |
|  |  |  |  |  |  |  |
| **Post-RT %Δ Monocytes** | 1.004 | 0.564 - 1.788 | 0.9888 | 1.504 | 0.868 - 2.608 | 0.1460 |
|  |  |  |  |  |  |  |

**Supplementary Figure 5.** Associations between Number of Radiation Fractions and Monocyte Counts.
